# DSCI: a database of synthetic biology components for innate immunity and cell engineering decision-making processes

**DOI:** 10.1007/s44307-024-00036-6

**Published:** 2024-09-03

**Authors:** Chenqiu Zhang, Tianjian Chen, Zhiyu Li, Qing Lu, Xiaotong Luo, Sihui Cai, Jie Zhou, Jian Ren, Jun Cui

**Affiliations:** 1 MOE Key Laboratory of Gene Function and Regulation, Guangdong Province Key Laboratory of Pharmaceutical Functional Genes, State Key Laboratory of Biocontrol, School of Life Sciences, Sun Yat-Sen University, Guangzhou, Guangdong 510275 China; 2 State Key Laboratory of Oncology in South China, Cancer Center, Collaborative Innovation Center for Cancer Medicine, School of Life Sciences, Sun Yat-sen University, Guangzhou, 510275 China; 3 State Key Laboratory of Membrane Biology & Frontier Research Center for Biological Structure, School of Life Sciences, Tsinghua University, Beijing, 100101 China; 4 Tsinghua University-Peking University Joint Center for Life Sciences, Beijing, 100101 China

**Keywords:** Database, Innate immunity, Synthetic biology, Interaction network, Signal motif, Loop visualization

## Abstract

Although significant progress of clinical strategy has been made in gene editing and cell engineering in immunotherapy, it is now apparent that design and modification in terms of complex signaling pathways and motifs on medical synthetic biology are still full of challenges. Innate immunity, the first line of host defense against pathogens, is critical for anti-pathogens immune response as well as regulating durable and protective T cell-mediated anti-tumor responses. Here, we introduce DSCI (Database of Synthetic Biology Components for Innate Immunity, https://dsci.renlab.cn/), a web-accessible and integrative database that provides better insights and strategies for innate immune signaling circuit design in biosynthesis. Users can interactively navigate comprehensive and carefully curated components resources that presented as visualized signaling motifs that participate in innate immunity. The current release of DSCI incorporates 1240 independent components and more than 4000 specific entries contextually annotated from public literature with experimental verification. The data integrated into DSCI includes the components of pathways, relationships between regulators, signal motifs based on regulatory cascades, and loop graphs, all of which have been comprehensively annotated to help guide modifications to gene circuits. With the support of DSCI, users can easily obtain guidance of gene circuits construction to make decision of cell engineering based on innate immunity. DSCI not only provides comprehensive and specialized resource on the biological components of innate immune synthesis, but also serves as a useful tool to offer modification or generation strategies for medical synthetic biology.

## Introduction

Synthetic biology is an emerging discipline that combines genetic tools with artificial design principles or assembles biological components to construct useful gene circuits, bringing new capabilities to medicine and pharmaceuticals (Cubillos-Ruiz et al. 2021; Yan et al. 2023; Ye and Fussenegger 2014). Two principles are considered in synthetic biology, including "bottle-up" and "top-down", referring to the assemble bio-components for de novo artificial life creation and the natural cell engineering to meet the needs of researchers respectively (Breithaupt 2006; Roberts et al. 2013; Yan et al. 2023; Zhou et al. 2020b). With the extensive development of relevant methods such as CRISPR/Cas9 system, next-generation sequencing and high-throughput screening technology, synthetic biology has expanded to the fields of medicine, drug development and chemical engineering, playing a role in combating diseases (Shalem et al. 2015). Researchers have created various novel strategies for cell engineering through gene circuits design for cancer treatment, such as chimeric antigen receptor (CAR)-T, T cell receptor (TCR)-T and CAR-M (Srivastava and Riddell 2015). These therapies can produce powerful anti-tumor therapeutic effects by modifying the patient's own immune cells and transferring them back to the patient (Feins et al. 2019). However, from a holistic perspective, the regulation of immune cells requires consideration of the synergistic effects of multiple factors, including cytokines rather than just artificial receptors, to achieve appropriate immune responses. Therefore, according to specific treatment needs, it is necessary to modify the entire signaling pathways of immune cells.

Innate immunity is the first line of host defense against pathogens. Invading pathogens can be recognized by pattern recognition receptors (PRRs), including Toll-like receptors (TLRs), NOD-like receptors (NLRs), RIG-I-like receptors (RLRs), and many other intracellular DNA and RNA sensors (Chen and Holtzman 2022; Karki and Kanneganti 2022; Takeuchi and Akira 2010). Upon recognizing pathogen-associated molecular patterns (PAMPs) or interacting with damage-associated molecular patterns (DAMPs), PRRs initiate a variety of signaling cascades, including type I interferon (IFN-I) signaling pathway, nuclear factor kappa B (NF-κB) signaling pathway and inflammasome pathway, thus promoting diverse proinflammatory or antiviral cytokines and chemokines which contribute to the downstream immune response activation. Signal cascades are composed with multiple components, including ligands, receptors, signaling proteins and regulator proteins. Regulated by transcription, post-translational modifications, positive or negative feedback and interaction of proteins, the components of the signaling pathway string and intertwine as a complex regulatory network (Tomalka et al. 2022). Once the balance of signaling pathways is disrupted, this complex and intricate immune network can be harmful, or even fatal. Therefore, introducing gene circuits into immune cells needs higher requirements and standards.

To better understand and apply synthetic biology methods to study and utilize the innate immune system, it is necessary to establish an information database and conduct analysis at the system level. Up to now, abundant but complicated data can be browsed in NCBI (Sayers et al. 2021; Schoch et al. 2020), UniProt (Consortium, 2023) and KEGG (Kanehisa et al. 2021, 2017), containing information about signaling pathway, protein function, protein localization, modification, protein structure and so on. Also, some specific immune databases like InnateDB (https://www.innatedb.com/) (Breuer et al. 2013; Lynn et al. 2008), Immport (https://www.immport.org/shared/home) (Bhattacharya et al. 2018) and VDJbase (https://vdjbase.org/) (Omer et al. 2020) provide transcriptomic and proteomic data from immune-relevant cells. Registry of standard biological parts (http://parts.igem.org/) founded with international genetically engineered machine (iGEM) competition (Vilanova and Porcar 2014; Warmbrod et al. 2020), synbioML (http://www.synbioml.org/) and ERMer (https://ermer.biodesign.ac.cn/) present as mature synthetic biology databases providing multiple services especially for microorganism (Mao et al. 2022). Additionally, a standard language for biosynthetic of components design and data transformation has been built, called synthetic biology open language (SBOL, https://sbolstandard.org/) (Baig et al. 2020; Bartley et al. 2015; Quinn et al. 2015). Although the databases mentioned above have greatly aided the research of synthetic biology or immune therapy, none of these resources provides complete components data or detailed regulatory layers for innate immunity that are essential for the design of immune signaling circuits. To further promote translational research on biomedicine, a standard biosynthetic database for innate immunity is needed.

In this study, we present DSCI (Database of Synthetic Biology Components for Innate Immunity, http://DSCI.renlab.cn), a novel bio-synthetic database for innate immunity that provides annotation, visualization, easily searchable component data and recommendations for gene circuit modification strategies. All data were collected from experimentally validated literature and divided into three different categories, including components, regulatory relationships, and pathways, to meet the requirements of accurate description of hierarchical signal networks. Due to the high complexity of multiple regulatory factors in innate immune regulation, the components of bio-systems under signal motifs presented on DSCI will bring different regulatory relationships together, helping researchers to understand the molecules of interest from different perspectives. Furthermore, DSCI also provides the loop data of innate immune signaling network to guide biologists to identify and effectively design gene circuits related to their studies. Compared to previous databases, DCSI provides modular components with interactions and modification information as well as the design strategies to aid the engineering of immune cells.

## Materials and methods

### Data collection, standardization and curation

All components, regulatory relationships and pathway data in DSCI were curated manually from literature. The literature mining was divided into three layers to ensure the completeness and diversity of data. The first layer was performed via retrieving in ‘*PubMed*’ (Fiorini et al. 2017) and ‘*Web of Science*’ (Chen et al. 2020) by key words: innate immunity, cytokine, inflammation and anti-virus, that these words can let us have an overview of the whole discipline. Then with the clear recognition of innate immunity and the demand of its bio-synthesis, we listed a series of information we needed for database construction, include signal proteins, regulatory relationship, interacting partner, regulator of signaling or not, modification type, modification site, executive enzyme, reference, constitutive or inducible expression, signaling function, stabilization or degradation, stimuli and bio-process, a total of 12 items. The second layer focused on the detailed regulatory relationship and retrieved by the general and specific key words: modification, ubiquitination, phosphorylation, SUMOylation, methylation, GlcNAcylation, acetylation, glutamylation, palmitoylation, degradation, stabilization, up-regulate, down-regulate, feedback loop and feed-forward loop etc. In this layer, we get the dominant data to form the signaling pathways with specific bio-process by our own, and add all the regulators in the pathway to make up the comprehensive regulatory network stringed by the regulatory relationship like modification or degradation. At the same time, we draw the loop map through literature to construct the smaller signal unit in the whole pathway. The third layer based on the signal proteins via searching key words: RIG-I, TBK1, IRF3, cGAS, STING, NF-κB, MyD88, TRAF6, IKKα and other molecules in total. So that, we ensured the integrity of the interaction data of these signal proteins, above all things, we established the signal motifs which take them as the center and present the comprehensive bio-process while they were activated.

In DSCI, a certain number of data obtained from experiment figures rather than the paragraph, such as the stability of protein, constitutive or inducible expression, and the modification sites. Stability of signaling proteins can be identified by western blot (WB) figures and its quantitative analysis, mainly functions in regulating the activation level of innate immunity (Goldenzweig and Fleishman 2018; Parzych and Klionsky 2014). Similarly, whether a protein is constitutive expressed or inducible expressed after stimulation can also be identified through WB or real-time quantitative polymerase chain reaction (RT-qPCR) analysis. Mass spectrometry (MS) analysis is also a reliable data resource for checking the modification types and sites (Buchberger et al. 2018). And we supplement protein annotations like gene name, protein name, basal function, sequence, localization, feature and so on, obtained from UniProt/NCBI.

### Database and web site implementation

All data in DSCI were stored and managed by MySQL tables. The server-backend development was based on Java and the web-frontend interfaces were implemented in Hyper Text Markup Language (HTML), Cascading Style Sheets (CSS) and JavaScript (JS). To provide better visualization for regulatory relationships and signaling network, network graphs were generated by Echarts on the website.

## Results

### Data curation of the components, regulatory relationships, and pathways of innate immune signaling network

**Fig. 1:**
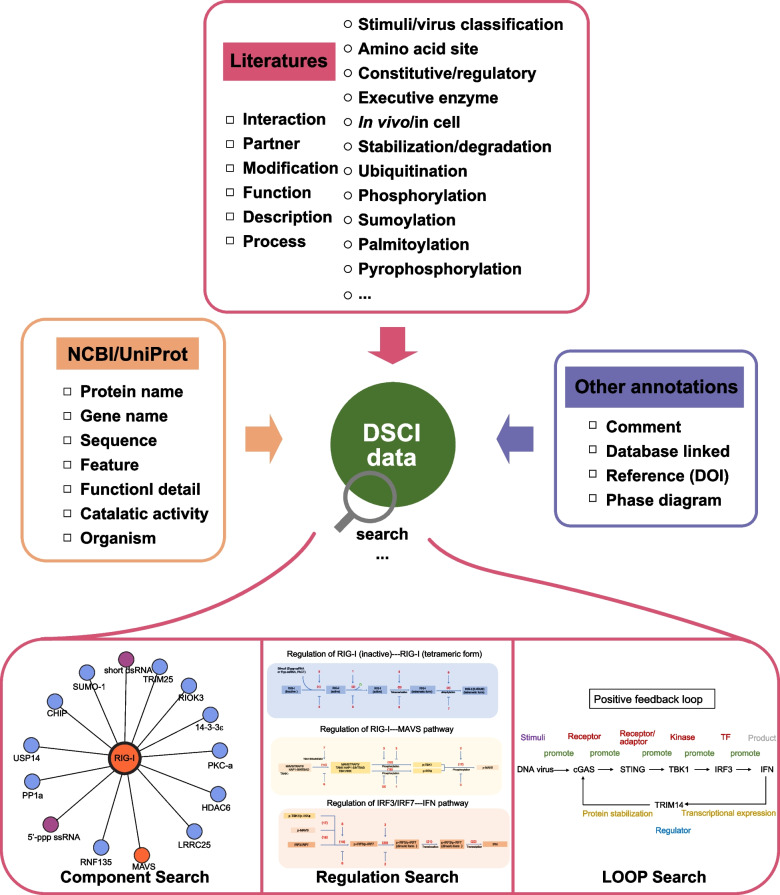
Data structure of DSCI. The data in DSCI are collected from literature then divided as information of main components to various items (as listed in the left column of the top box) and the more detailed sub-level classification (as listed in the right column of the top box). Certain protein name, sequence and important annotations are from NCBI (Schoch et al. 2020) and UniProt (Consortium, 2023) (box on the left). Other annotations are added to organize data methodically (box on the right). DCSI provides three kinds of functional search mode, including Component search, Regulation search and LOOP search

As a novel platform of synthetic biology for innate immunity, DSCI records component data from existing literature and annotates it according to the other resources to support multiple types of retrieval modes (Fig. 1). Furthermore, we classify these data into three layer of datasets classification including components, regulatory relationships, and pathways, are divided into 11 more detailed sections.

The information of components from innate immune signaling network are all collected from assessable literature with experimental support. To differentiate these components, we have categorized them as regulators, signaling proteins and stimuli, including over 1200 independent components in total. Signaling proteins, such as receptors, adaptors, enzymes, and transcriptional factors, are the main components of signaling pathways, which function in signaling transduction and signaling cascade. Stimuli, representing the exogenous pathogens or endogenous biological macromolecules such as DNA/RNA (Zindel and Kubes 2020), are the ligands of signaling pathway by targeting signal proteins to trigger signaling pathways (Xue et al. 2019). Regulators are the proteins that mediate the function of signal proteins to up-regulate or down-regulate the signaling transduction. Signaling proteins, stimuli and regulators together form the innate immune regulatory network.

Regulatory relationship strings various components together to form a new set. Different with previous databases, the regulatory relationship section in our database records not only the information of protein interaction but also additional information like modification and function, and imparting directionality to the connections between different components. For modification section, we identify and define 24 different modification types as subsets, and record 597 modification sites that extracted from diverse research evidence. Besides the common protein modifications, we also pay close attention to certain novel protein modifications, such as pyrophosphorylation (Yang et al. 2023), S-nitrosation (Liu et al. 2021) and ATG8ylation (Nguyen et al. 2021). These modification subsets contain less than 4 pieces of data due to limited knowledge of their function in innate immunity, compared with hundred pieces of data in ubiquitination or phosphorylation subsets, indicating the potential for research on the mechanisms and functions of these novel modifications. For the function of regulatory relationship, we obtain at least 1144 impact results in signaling pathway based on the experimental evidence from literature, and cover over 219 events which are related to protein stability.

The pathway section is comprised by signaling proteins which represent signal transmission regulated by regulators. Each signaling protein acts as a signal node and form a function motif with its regulators. Signal motifs not only include relatively independent signal transmission mode but also have clear chemical reaction process. Here, we set 32 signal motifs which include 124 bio-processes and 571 enzymes working relationships (Table 1).

**Table 1:** Overview of the data subsets in DSCI

Classification	Data type	Number of entry
Component	Regulator	1148
	Signaling protein	74
	Stimuli	18
Regulatory Relationship	Functional direction	1144
	Enzyme & Partner	571
	Modification site	597
	Modification type	24
	Protein stabilization	219
Pathway	Signal motif	32
	Bio-reaction process	124
	Modification working network	54

### User-friendly and diverse web interface of DSCI

**Fig. 2:**
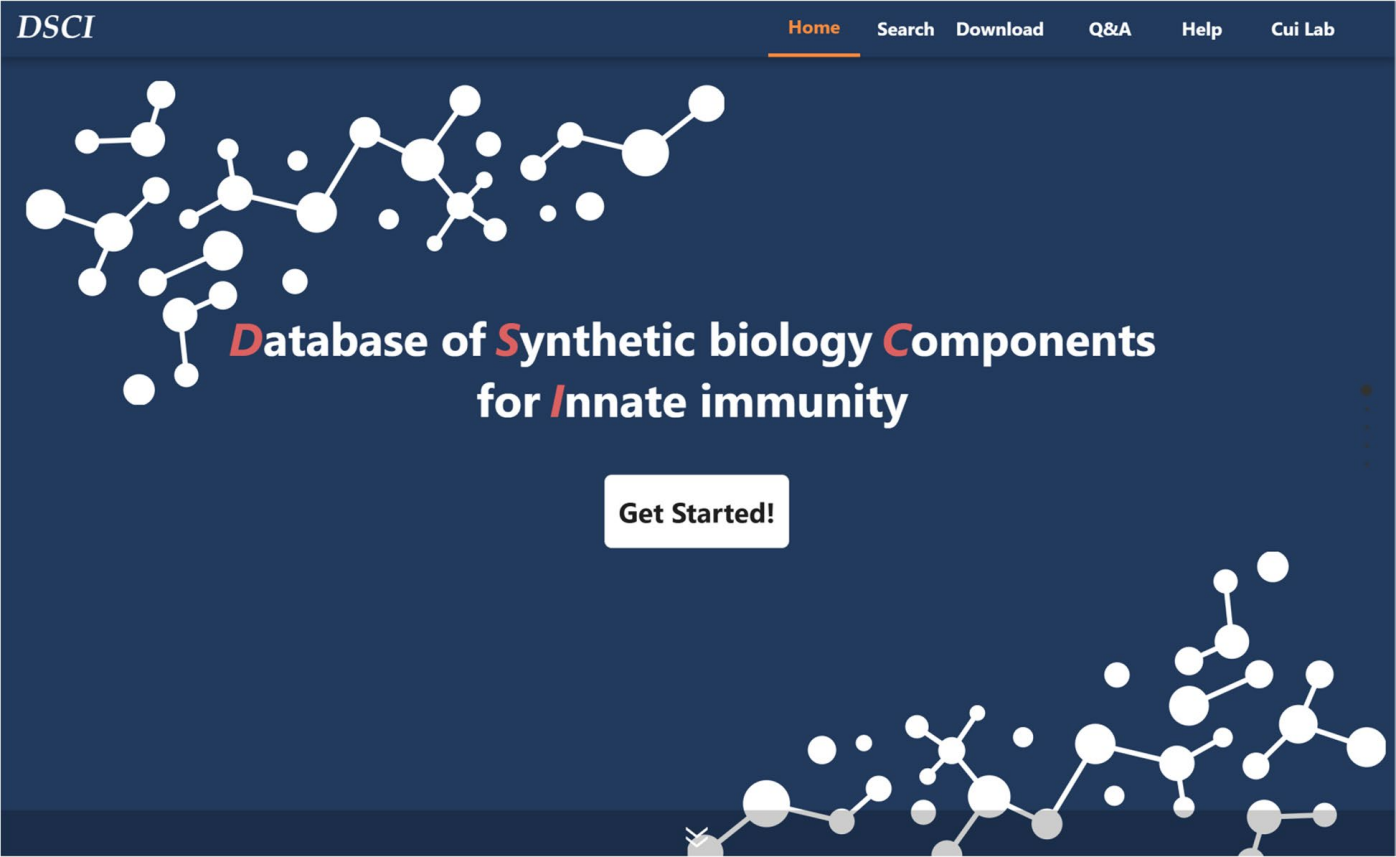
A screenshot of the home page of DSCI. Each functional block is shown on the upper right corner of the page, and supporting quickly search with the simple search bar in the middle which links to the search details page. The home page also provides further information on each search mode including database introduction, data type and working scene at the bottom of the page

The user-friendly web interfaces of DSCI allow users to search, download and ask questions conveniently. This interface has been developed with experimental biologists and web engineer in close cooperation to ensure the intuitive and easy to use for the biologists in both simple and more advanced searching. The database includes *Home*, *Search*, *Q&A*, *Help* and *Download* block (Fig. 2).

**Fig. 3:**
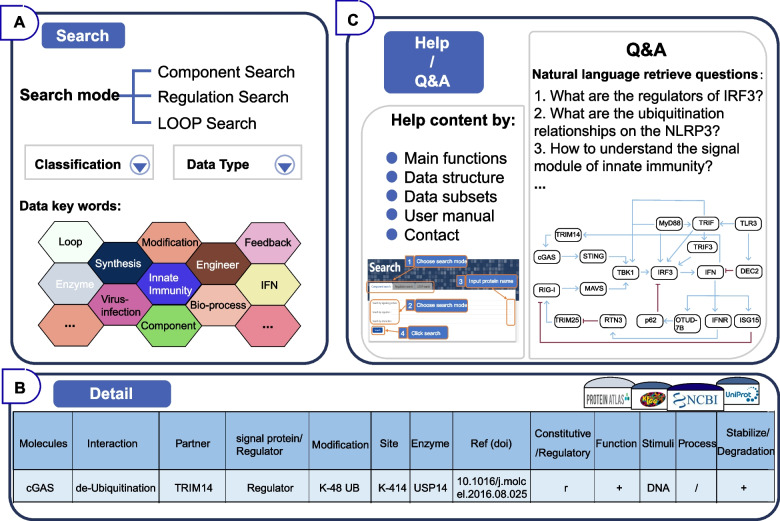
Basic functions of the DSCI web interface. **A** The main pattern of the search mode of DSCI. **B** The detail interface of protein information jumping from clicking node stands for TRIM14. **C** Q&A and Help interface for better utilize of the DSCI

From the "Search" block that serves as the core of DSCI, users can choose "Component search", "Regulation search", and "LOOP search" modes, and choose more detailed classifications and data types. Each specific search page allows one to search molecule of interest either by gene name or protein name easily through a fuzzy search that locates likely relevant to argument. DSCI records lists of genes and proteins name frequently known or previously been known from Uniprot and NCBI, that automatically present possible objects for query. Through the classification and data type screening, various key words accurately describe the suitable innate immune information for biosynthesis (Fig. 3A).

"Component search" is focused on specific component and enables interactive exploration of the interaction map with accurate regulation relationships. DSCI shows all relevant regulatory interactions with lines and components with nodes, which are stringed together and specifically labeled. In addition, users can add extra query component for advanced interaction network or pick out data type like modification of interest for exploration. In this way, the users can explore deeper regulation relationship between signaling pathways and exclude specific interactions. Besides, users can choose any node or line in the graph for the detail information, including gene name, function, modification site, references, and catalytic activity which are collected from literature with experimental validation (Fig. 3B).

The "Regulation search" page provides signal motifs which provides signal transduction with detailed chemical processes in a smaller unit rather than the whole complex signaling network. The signal motif supports enormous flexibility in searching, analysis and understanding of components and their interaction of interest. Users can choose a specific component to search its interaction partners and its function in signaling pathway, or select multiple components retrieval to find the potential relationship among them in the signaling pathway. In addition, the bio-process data in specific signal motif help analyze the kinetic change under different combinations of reactions.

The "LOOP search" contains feedback and feed-forward loops which are essential for cells to exert appropriate immune responses. They are widely and usefully applicated in biosynthetic pathways to regulate key enzyme’s activity by corresponding end-products (Gödecke et al. 2020; Michalska et al. 2018; Park et al. 2007). Similarly, the loop relationship recorded here has been standardized to provide a reliable source of gene circuits synthesis. Users can search with components, regulatory relationship and product of a pathway of interest. From the "LOOP search" results, detailed information of each component and interaction are included. Moreover, the links to the original supporting publication can be obtained.

"Q&A" block is implemented to retrieve answers associated in a natural language way, which can provide information to various questions about synthetic biology and innate immune signaling network. Several questions are predefined in DSCI to demonstrate the convenient and power of application guidance of engineering innate immune signaling pathways. For example, for the question "How to maintain the IFN level appropriately through IRF3-mediated transcription of downstream genes?", it can be searched in "Component search" with the transcriptional factor "IRF3" and get a graph which present all related regulators and their functions. Besides, users can also search "IRF3" in "Regulation search" to receive the signal motif that IRF3 may participate. Furthermore, the users can search in "LOOP search" mode with key words "IFN", perhaps with "IRF3" as supplement, and chose "Negative feedback loop" in loop type to get a negative feedback loop containing indicated component we collected. The level of description detail is related to the accuracy of the result (Fig. 3C, right).

"Help" block shows a tutorial to use DSCI database, and lists content in accordance with "Main functions", "Data structure", "Data subsets" and "Contact". Users are also allowed to get the detailed user guide which explains how to reproduce the results of case studies with screen shots taken step by step from the help page (Fig. 3C, left).

"Download" block encompasses all data within the DSCI in standardized format. Additionally, a user manual is available for download on this page.

### Robust visualizing and regulatory landscape for signaling pathways through graph database

**Fig. 4:**
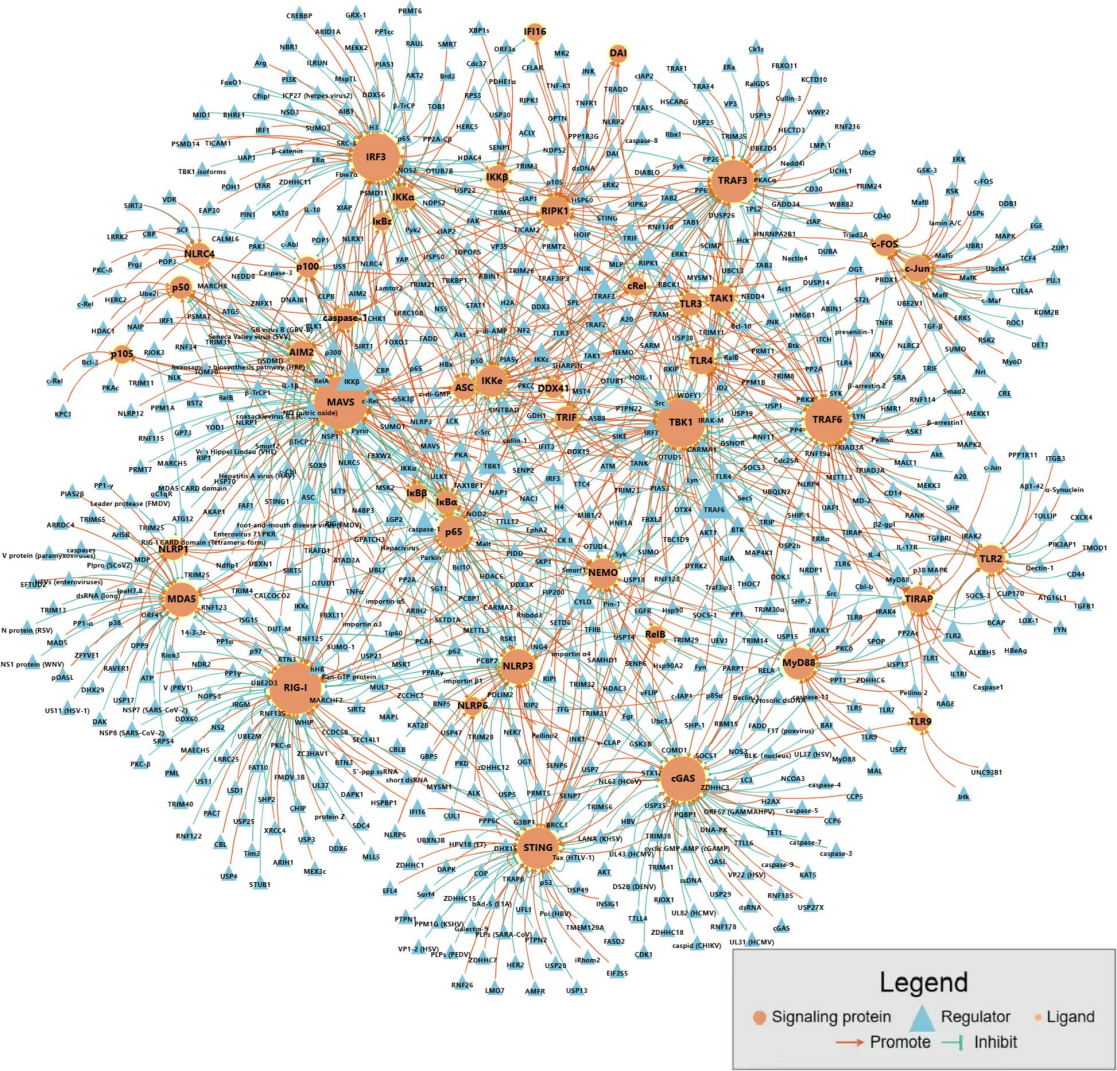
Component network of innate immune signaling pathway. In order to achieve comparison of specific component interaction relationship side-by-side, an interactive exploration is used to show all relevant components and their interactions for a query subject. Nodes (i.e. component) and lines (i.e. interaction) are colored to distinguish the property of components. By right clicking on a node, you can interactively link to the detailed page in DSCI to obtain more annotations about the components of interest

The majority of component and interaction data available in other databases primarily refers to independent genes and proteins. However, DSCI is designed to visualize the components and regulatory relationships of innate immune signaling network in interaction map which enables to guide engineering of gene circuits for immune cells (Fig. 4).

**Fig. 5:**
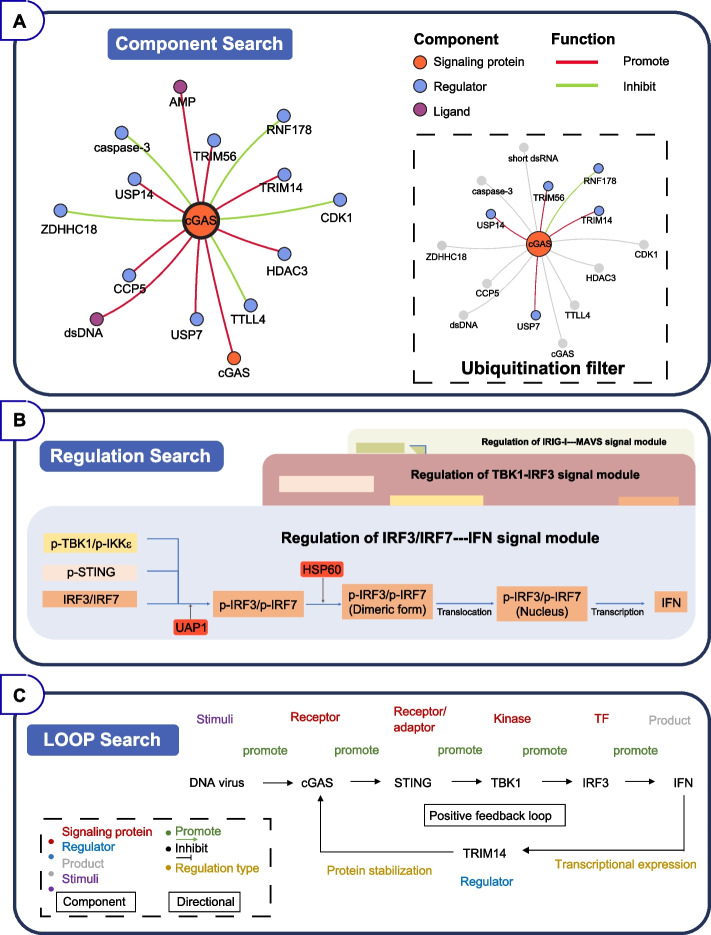
The diagram of Component search, Regulation search and LOOP search. **A** The interface of Component search in "cGAS" and filter with ubiquitination condition. **B** The interface of Regulation search mode with signal motifs for UAP1 and HSP60 regulatory relationship in DSCI. **C** The LOOP search interface of bio-synthesis guiding retrieval

Here, we illustrate an example to show how to utilize DSCI by "Search" block. We investigated the components of innate immune signaling pathway in DNA virus infection and got a suggestion on how to enhance antiviral ability using DSCI. In the search box of home page or "Component search" page, users can check whether specific component of interest participates in antiviral responses. Take cGAS as an example. cGAS is a DNA sensor that senses both exogenous and endogenous DNA, and participates in various innate immune signaling including IFN-I signaling and NF-κB signaling (Sun et al. 2013). We can acquire relevant regulatory interactions of cGAS presented as a graph along with functional labels in the result page of "Component search" mode, and the segments that generally point to the center node showing the direction and function of their interactions (Fig. 5A, left map). In addition, the "Component search" mode allows users to get more distinct graph like modifications, partners, or functions by adding indicated search condition. If the users want to find the molecules that affect the ubiquitination of cGAS, they can add modification feature or input "ubiquitination" to filter the results, and all the molecules responsible for cGAS ubiquitination could be shown (Fig. 5A, right map).

The "Regulation search" mode supports searching for the specific regulators through signal motifs within particular pathway of interest. For example, IRF3 functions as a critical protein in IFN-I signaling pathway, its phosphorylation is essential in signaling activation. Lots of kinase are identified to facilitates the phosphorylation of IRF3. UAP1, a metabolic enzyme, is identified to catalyze the pyrophosphorylation of IRF3 on serine (Ser) 386 to promote the phosphorylation of Ser 396 (Yang et al. 2023). Additionally, HSP60 is also reported to interact with IRF3 to facilitate its phosphorylation and dimerization (Lin et al. 2014). In traditional databases, users can only get the information that both UAP1 and HSP60 can interact with IRF3 and regulate its phosphorylation, which may cause the misleading that UAP1 and HSP60 work in the same manner. However, the "Regulation search" mode in DSCI divides different regulatory relationships and signal layers of components in more detail. Users can not only get the difference of the mechanism of IRF3 mediated by UAP1 or HSP60, but also obtain the working sequence of these molecules during signaling transduction (Fig. 5B).

The combination of feedback and feed-forward loops in signaling network can result in complex dynamic phenomena such as bio-switches and oscillations to regulate immune response accurately (Slepchenko and Terasaki 2004; Tyson et al. 2003). The "LOOP search" supports searching for feedback and feed-forward loops involving relevant molecules and products of interest in innate immune signaling network. Using the information automatically extracted from DSCI, a localization-based loop map layout can be loaded. Under "LOOP search" mode, users can search the key words one by one or add together, and get the filtered loop graph after clicking the "Search" button. Taking the production of IFN-I as an example, if users want to find a strategy to enhance their antiviral ability, many processes can be done to meet this demand, such as regulating the protein modification, gene transcription, protein stability, etc. By using "LOOP search", a positive feedback loop containing TRIM14 and cGAS could be found (Fig. 5C), showing that IFN-induced transcription of TRIM14 may lead to the de-ubiquitination and stabilization of cGAS, promote the signaling transduction in IFN-I signaling (Chen et al. 2016). Since this positive feedback loop contains various physiological process to facilitate IFN production, it can also be a suggested strategy for enhancing antiviral response.

### Exploring loop data in an interaction network context

**Fig. 6:**
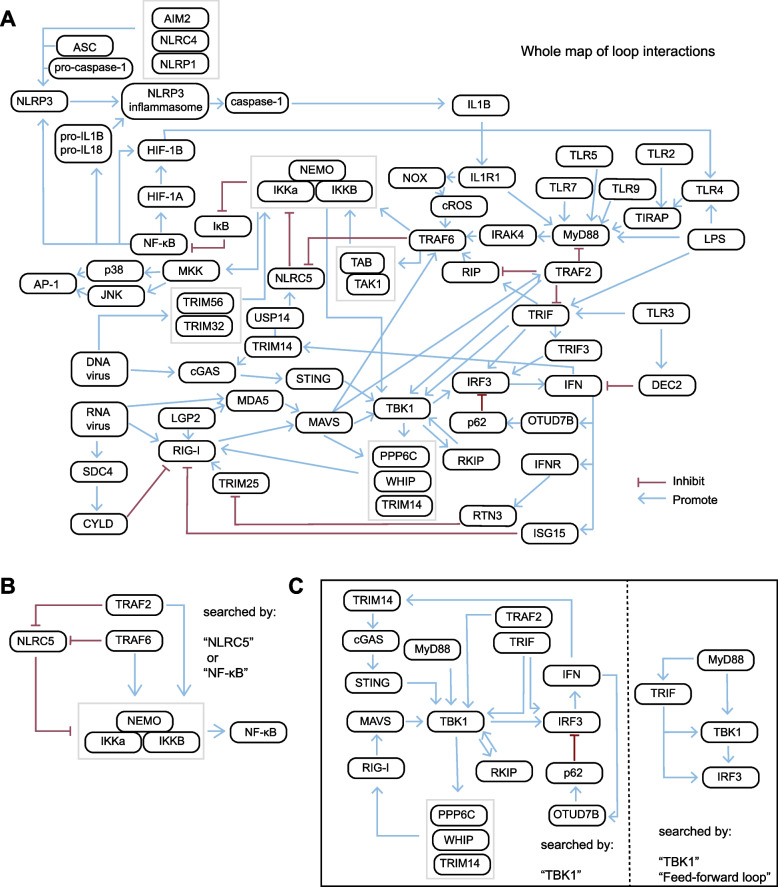
Construction and guidance of multi-level loop map. **A** The whole map of multi-level loop relations that are identified with experimentally validated literature recorded in DSCI which supports automatic generation regarding to the demand gene circuits. **B** The loop map searched by the key words "NF-κB" and "NLRC5". **C** The loop map searched by the key words "TBK1" (left map), and searched by the key words "TBK1" along with “feed-forward loop” (left map)

DSCI also provides the whole map of feedback and feed-forward loops within innate immune signaling networks with multiple key words (Fig. 6A). Feedback and feed-forward loops are sort of system termed "bistable" that effectively control hierarchical inputs into switch-like and irreversible responses, has developed great prospects in biosynthesis for highly robust and reliable output (Ferrell 2002; Folliard et al. 2017; Matsu-Ura et al. 2018). We previously reported an investigation of NLRC5 modulating NF-κB activation through reversible ubiquitination with experiments and mathematical modeling, uncovered cellular heterogeneity to NF-κB activation in response to NLRC5 ablation, that identified a coherent feed-forward loop generated by NLRC5 ubiquitination to explain its role in the dynamic control of innate immunity (Meng et al. 2015). In the original paper, the signal loop pattern is identified through manual assignment based on many different literatures, but DSCI enables various loop strategies of demand retrieval in less than a few seconds. As a case example of DSCI, users can search with the key words "NLRC5" or "NF-κB" in "LOOP search" mode to get the information of the loop map mentioned above (Fig. 6B).

In addition, users can describe and input their own needs and get different results of specific information about loops within innate immune signaling network. For example, with the single component "TBK1" search in "LOOP search" mode, users can get the whole loop map containing TBK1with abundant information. In this way, various loop strategies that indirectly related to TBK1 are also provided, such as the transcriptional factor IRF3 inhibition due to its own ubiquitination (Xie et al. 2022), and the receptor RIG-I activation based on its own phosphorylation (Tan et al. 2017) etc*.* (Fig. 6C, left map). If users only focus on specific loop type like feed-forward loop, they can add key word "feed-forward loop" as a restriction to filter the results. Once the users extract feed-forward loop with TBK1, they will get more specific results showing the dual feed-forward loop modulate IFN-I responses during TLR4 activation with TBK1 (Zhou et al. 2020a) (Fig. 6C, right map). With the strategies recommended in "LOOP search" mode, users can obtain the gene circuits design and modification strategies to apply on cell engineering. The modification on these feedback and feed-forward loops will amplify the potential of precise and in time regulation of signal pathway activation. Detailed tutorial to the cell engineering guiding process has been developed and is available at "HELP" page.

## Discussion

Due to the rapid development of synthetic biology in the therapy of immune disorders disease, it is necessary to integrate protein composition data of innate immune signaling network to guide cell engineering. Similar synthesis biology databases have been constructed for different purpose. Registry of standard biological parts founded with iGEM competition, synbioML and ERMer collect and provide multiple genetic parts among all species, to benefit the development of synthetic biology devices and systems. InnateDB, Immport and VDJbase focus on immunity data and provide abundant multi-omics information in human, mouse and some other species, which systematically integrate the knowledge of immune signaling regulation network. However, these databases are complex and difficult to start with, visualized data are incomplete and hard to obtain, independent bioinformatic resources without standardization and systematic analysis also cause difficulties for gene circuits design and cells engineering in innate immunity related biosynthesis. To improve and perfect the guidance of innate immunity synthetic biology, we constructed DSCI, a novel database of the innate immunity components for synthetic biology that provides standardized components and regulatory relationship in signal motifs for guiding cell engineering. More than 4000 entries of data have been recorded in DSCI with manual curation, through the review of approximately 5000 journal articles. Signal motif and loop map of signaling pathway in DSCI have a huge potential to elucidate the relationship between components and guide the engineering of immune signaling pathways. Meanwhile, standard format record in DSCI have great potential to develop tools for data utilization, including but not limited to target prediction (Luo et al. 2022; Varadi et al. 2022), signaling imitation (Babur et al. 2021), data enrichment analysis (Aleksander et al. 2023; Consortium, 2015) and alignment search (O'Driscoll et al. 2015). To present data for easy understanding, we also constructed accessible visualized figure that essential for the design of immune signaling circuits in DSCI. In conclusion, DSCI is a convenient database with easy operation system as well as wide-range and accurate data information in innate immunity, which provides better insight and strategies for innate immune signaling circuit design in biosynthesis to benefits users.

DSCI has the following advantages in comparison with traditional immunological information database: (i) DSCI integrates the most comprehensive components information through hierarchical literature mining and divided these data into 3 classification includes component, regulatory relationships, and pathways, and 11 data type to support different search modes. (ii) DSCI provides unique retrieval modes of "Regulation search" and "LOOP search" dedicated to guiding synthetic biology through recommend gene circuits design and modification strategies. (iii) DSCI has user-friendly web interfaces which support easy retrieval and provide various interactive diagrams, which users can browse all the components and search interested data by various criteria. (iv) DSCI is built into a structure that can be further upgraded, ensuring update regularly through scanning newly published literatures and data submissions from the research community.

In summary, DSCI provides useful information on innate immune components for synthetic biology through literature search and experimental verification, and it could guide biologists for cell engineering strategies. DSCI will maintain long-term updates by scanning newly published literature and other database information in public databases. More analysis functions about innate immune signaling networks will also be included to further improve our application framework.

## Data Availability

Raw data of the DSCI (http://DSCI.renlab.cn) have been deposited in the database. The data that support this study are available within the article and its Supplementary Information files or available from the authors upon request. Data generated in this study will be made available from the Lead Contact for academic/non-commercial research purposes on request under a Material Transfer Agreement.

## Abbreviations

CAR: Chimeric antigen receptor
cGAS: Cyclic GMP-AMP synthase
CSS: Cascading style sheets
DAMPs: Damage-associated molecular patterns
DSCI: Database of Synthetic Biology Components for Innate Immunity
HSP60: Heat shock protein 60
HTML: Hyper text markup language
IFN-I: Type I interferon
IKKα: The inhibitor of kappa-B kinase alpha
IRF3: Interferon regulatory factor 3
JS: Javascript
MyD88: Myeloid differentiation primary response protein 88
NF-κB: Nuclear factor kappa B
NLRC5: NLR family card domain containing 5
NLRs: NOD-like receptors
PAMPs: Pathogen-associated molecular patterns
PRRs: Pattern recognition receptors
PTM: Post-translation modification
RLRs: RIG-I-like receptors
Ser: Serine
STING: Stimulator of interferon genes
SUMO: Small ubiquitin-like modifier
TBK1: TANK-binding kinase 1
TCR: T cell receptor
TLRs: Toll-like receptors
TRAF6: TNF receptor associated factor 6
TRIM14: Tripartite motif 14
UAP1: UDP-N-acetylglucosamine pyrophosphorylase 1
